# Antiretroviral Therapy Switch in HIV-Infected Adults from a Regional HIV/AIDS Center in NE Romania

**DOI:** 10.3390/medicina60060854

**Published:** 2024-05-24

**Authors:** Isabela Ioana Loghin, Șerban Alin Rusu, Andrei Vâţă, Mihaela Cobaschi, Ion Cecan, Carmen Manciuc, Carmen Mihaela Dorobăţ

**Affiliations:** 1 Department of Infectious Diseases, “Grigore T. Popa” University of Medicine and Pharmacy, 700115 Iasi, Romania; isabelabegezsan@yahoo.com (I.I.L.); dmanciuc@yahoo.com (C.M.); 2 Department of Infectious Diseases, “St. Parascheva” Clinical Hospital of Infectious Diseases, 700116 Iasi, Romania; rususerbanalin@yahoo.com (Ș.A.R.); ion11cecan@gmail.com (I.C.); carmendorobat@yahoo.com (C.M.D.); 3 Faculty of Medicine/Clinical II Department, “Carol Davila” University of Medicine and Pharmacy, 050474 Bucharest, Romania; 4 National Institute for Infectious Diseases “Prof. Dr. Matei Balș”, 021105 Bucharest, Romania

**Keywords:** HIV infection, ART switch, HIV-AIDS, treatment-experienced patients

## Abstract

*Background and Objectives*: Antiretroviral therapy (ART) has revolutionized the management of HIV infection, transforming it from a once-debilitating disease to a chronic, manageable condition. However, challenges such as treatment resistance, medication side effects, and long-term tolerability persist, prompting the exploration of novel therapeutic approaches. We aimed to highlight the characteristics and related comorbidities of HIV/AIDS cases in which the antiretroviral therapy was modified. *Material and Methods*: A cross-sectional clinical investigation was conducted on adults diagnosed with HIV/AIDS who were hospitalized at the “St. Parascheva” Clinical Hospital of Infectious Diseases in Iasi in the Northeastern region of Romania. The timeframe under investigation was 1 January 2023 to 30 June 2023. *Results*: In the Northeastern part of Romania, from a total of 1692 patients in the active records, there were a total of 148 recorded cases of antiretroviral therapy switch in HIV-infected patients. The main reason for the ART switch was the simplification of the ART regimen (82 cases, 55.40%), viro-immunological failure (16 cases, 10.66%), other disturbances correlated to the ART regimen, dyslipidemia (34 cases 22.97%), depression (3 cases, 2.02%), suicide attempt (1 case, 0.67%), new situations, including the appearance of pregnancy (3 cases 2.02%), and tuberculosis (9 cases, 6.08%). ART before the switch was represented by protease inhibitors that accounted for 84 cases (56.75%) of the ART switch. Following the therapy switch, integrase inhibitor-based ART single-tablet regimens accounted for 43.91% (65 cases) of all changeovers, with non-nucleoside reverse transcriptase inhibitor regimens coming in second, in 63 cases, 42.66%. *Conclusions*: ART switch as an experimental therapy offers a promising approach to optimizing HIV treatment outcomes. By focusing on viral suppression and immune reconstitution, addressing treatment challenges, and exploring novel ARV agents, ART switch strategies aim to improve the overall health and well-being of individuals living with HIV.

## 1. Introduction

Antiretroviral therapy (ART) has revolutionized the management of HIV infection, transforming it from a once-debilitating disease to a chronic, manageable condition. However, challenges such as treatment resistance, medication side effects, and long-term tolerability persist, prompting the exploration of novel therapeutic approaches. In recent years, deliberate antiretroviral (ART) regimen switching has emerged as an intriguing strategy to optimize treatment outcomes for individuals living with HIV [1,2].

One of the primary goals of ART switch strategies is to optimize viral suppression, which is crucial for controlling HIV replication and preventing disease progression. Studies have demonstrated that a strategic switch from one ART regimen to another can lead to a significant reduction in viral load, thereby enhancing treatment efficacy. By targeting distinct mechanisms of action and overcoming resistance pathways, ART switches offer a renewed opportunity to achieve and maintain the durable suppression of the virus [3,4].

In addition to viral suppression, ART switch interventions have been associated with improvements in immune reconstitution. Research has indicated that switching ART regimens can result in notable increases in CD4 cell counts, reflecting a restoration of immune function and resilience against opportunistic infections. This immune recovery is essential for halting disease progression and improving the overall health outcomes in individuals living with HIV [5,6].

ART switch strategies also provide solutions to challenges related to treatment adherence and medication tolerability. Some patients may experience adverse effects or difficulties adhering to their current regimen, leading to suboptimal treatment outcomes. By switching to alternative ARV drugs, patients may experience reduced side effects, improved adherence, and an enhanced quality of life [7,8].

The requirement of adjusting HIV treatment according to the needs of each patient has been highlighted by the era of customized medicine. In order to determine the best regimens depending on variables like the virus subtype, treatment history, and comorbidities, comparative efficacy studies have assessed a variety of ART switch techniques. These studies have provided valuable insights into the efficacy and safety profiles of different switch options, enabling clinicians to make informed and personalized treatment decisions [9,10].

Safety considerations are paramount when implementing ART switch strategies. Research has focused on evaluating the safety profiles of different ART regimens, including potential risks such as drug interactions, metabolic disturbances, and cardiovascular events. Clinicians must carefully assess the benefits and risks of ART switch interventions to ensure the long-term success of treatment while minimizing adverse effects [5]. An important aspect is pre-conception counseling in couples with prospective ART modification. In this situation, the guidelines for treatment-naïve pregnant patients recommend regimens that include ritonavir-boosted atazanavir (ATV/r), darunavir (DRV/r), or raltegravir (RAL), in combination with one of the following NRTI backbones: abacavir (ABC)/lamivudine (3TC), tenofovir disoproxil fumarate (TDF)/emtricitabine (FTC), TDF/3TC, or AZT/3TC [1,11,12].

Emerging research has highlighted the potential use of novel ARV agents in ART switch strategies. Investigational drugs such as long-acting injectable formulations and novel classes of ARVs offer the promise of improved treatment adherence and a reduced pill burden [13,14]. 

Furthermore, the creation of medications with distinct modes of action opens up new possibilities for treating HIV patients and overcoming treatment resistance [14,15].

Effective patient education and support programs play a vital role in the success of ART switch strategies. Studies have shown that comprehensive patient counseling, adherence support, and regular follow-ups significantly enhance treatment outcomes and patient satisfaction. Empowering patients with knowledge about their treatment options and the potential side effects can lead to improved treatment adherence and overall well-being.

ART switch, as an experimental therapy, offers a promising approach to optimizing HIV treatment outcomes. By focusing on viral suppression and immune reconstitution, addressing treatment challenges, and exploring novel ARV agents, we aim to observe the evolution of the study group after the ART switch so that we can improve the overall health and well-being of individuals living with HIV.

## 2. Materials and Methods

### 2.1. Database Description

To highlight the characteristics and related comorbidities of HIV/AIDS cases in which the antiretroviral therapy was modified, we conducted a cross-sectional clinical investigation of patients diagnosed with HIV/AIDS who were hospitalized at the “Sf. Parascheva” Clinical Hospital of Infectious Diseases in Iasi in the Northeastern region of Romania. The timeframe under investigation was 1 January 2023 to 30 June 2023.

The study included 148 patients who were admitted to our Regional HIV/AIDS Center of Northeastern Romania, were diagnosed with HIV/AIDS, had a positive test for HIV using the enzyme-linked immunosorbent assay (ELISA) and had the disease confirmed by western blot (WB), were under different antiretroviral regimens and were evaluated for switching therapy. In addition, the patients’ CD4+ T cell counts and HIV plasma viral load were evaluated. 

### 2.2. Ethical Approval

Ethical approval was obtained from the “St. Parascheva” Clinical Hospital of Infectious Diseases in Iasi, Romania, according to the Ethical Committee in March/2024; Approval No. 5/21. At admission, every person signed the informed consent form.

### 2.3. Study Design

Data on demographics specific to age and gender, individual pathological histories, clinical characteristics, blood tests (viro-immunological testing), assessments of possible opportunistic infections, patient staging, the initiation of antiretroviral therapy, and the course and prognosis of HIV/AIDS were all collected. Because of this type of design, this study is classified as a cross-sectional study.

The HIV infection stage was identified using an age-specific CD4+ T-lymphocyte count or the CD4+ T-lymphocyte percentage of the total CD4 T-lymphocyte cell level, according to the Centers for Disease Control and Prevention (CDC) in Atlanta. Three phases are distinguished between HIV infection and AIDS: stage 1, characterized by CD4+ T-lymphocyte levels greater than 500 cells/mL; stage 2, characterized by levels between 200 and 499 cells/mL; and stage 3, characterized by levels less than 200 cells/mL. Stages 1 and 2 depict HIV infection, whereas Stage 3 represents AIDS (https://www.cdc.gov/hiv/library/reports/hiv-surveillance/vol-26-no-2/content/technical-notes.html (accessed on 15 January 2023)). ART switch was made according to the Liverpool drug–drug interaction checker, to avoid drug–drug interactions if the patient was taking other co-medication (https://www.hiv-druginteractions.org/checker (accessed on 1 February 2023)).

The adherence and therapeutic compliance were monitored in order to establish the necessity of switching the ART. 

### 2.4. Study Setting and Data Collection

The “St. Parascheva” Clinical Hospital of Infectious Disease in Iasi is a primary referral medical facility for the Moldova region of Romania, with a capacity of 300 beds. It is divided into six pavilions. This includes a compartment for Infectious Diseases and the HIV/AIDS Regional Center. The Regional Center has a capacity of 12 beds, where the patients are periodically evaluated based on CDC and EACS recommendations. 

All blood tests were performed by the hospital’s central laboratory, and the patient’s HIV plasmatic viral load (RT-PCR HIV 1 using Cepheid’s GeneXpert^®^ (Cepheid, CA, USA)) and CD4+ T cell count were assessed by the molecular biology lab. The viral load was classified as low if undetectable or with less than 40 copies/mL, and high if detectable with more than 40 copies/mL.

Periodically, the clinical and biological status of every newly diagnosed PLWH (people living with HIV) was assessed for metabolic syndrome and liver enzymes. The laboratory reference values were within 5–31 UI/L for AST (aspartate trans-aminase) and for ALT (alanine transaminase), within 7–32 UI/L for GGT (gamma-glutamyl transferase), within 122–200 mg/dL for COL (cholesterol), within 30–159 mg/dL for LDL-COL (low-density lipoprotein cholesterol), within 40–66 mg/dL for HDL-COL (high-density lipoprotein cholesterol), and within 30–150 mg/dL for TG (triglycerides), with no differences between sexes.

There are 1710 patients in total on the active register of the Regional HIV/AIDS Center, Iasi. Every six months, they receive a periodic evaluation to make sure they are adhering to and complying with the antiretroviral treatment. Every patient has a medical file that includes information on their related conditions, blood test results, CD4 T cell count, HIV viral load, and ART regimens. The paper charts kept by our center’s patients provided the data for our investigation.

### 2.5. Statistical Analysis

The analysis included the patients’ demographic data, medical history, clinical and laboratory results, treatment administered, and evolution.

The Chi square test in the XLSTAT version 2019 program was used to determine the correlation between demographic characteristics and clinical and laboratory data. Statistical Software for Excel (XLSTAT) version 2019 was used to conduct the statistical analysis. T-test statistics were used to quantify the difference between two data samples selected. *p*-value test statistics were performed, obtaining significant values under 0.05. All the data were analyzed, and on this basis, the statistic was made. No data were omitted.

## 3. Results

### 3.1. General Characteristics

From the 1710 patients in the active records of the Regional HIV/AIDS Department in the Northeastern part of Romania, there were a total of 148 recorded cases of antiretroviral therapy switch. Men were the ones who experienced more antiretroviral therapy switches (96 cases, 64.86%), and the difference in the M/F ratio between those with ART switch and those without was significant (1.23 vs. 1.84, *p* = 0.025). The study group’s median age was 35 years old, significantly lower than that of the patients without a therapy switch (40 years, *p* = 0.046). The age distribution was as follows: young adults, aged between 30–39 years old—85 patients (57.43%); 40–49—19 patients (12.83%); 50–59 years—17 patients (11.48%); 20–29 years—20 patients (13.50%); and over 60 years old—7 patients (4.79%) (Table 1). 

At the time of switch, 54 cases (36.48%) had a CD4+ T-lymphocyte level between 1 and 199 cells/μL, 52 cases (35.13%) had a CD4+ T-lymphocyte value between 200 and 499 cells/μL, and 42 cases (28.37%) had a value over 500 cells/μL. The mean CD4+ T-lymphocyte level was 330.95 cells/μL, with a standard deviation of 284.22. Based on both gender and the CD4+ T-lymphocyte levels, men were the most affected, with lower overall CD4+ T-lymphocyte levels. The average HIV viral load was 494.627 copies/mL, with a standard deviation of 109.703.

The following findings were obtained using the CDC (Center for Disease Control and Prevention) phases of HIV/AIDS. According to our data, 28 patients (18.91%) had stage 1 HIV infection, 52 patients (35.13%) had stage 2 HIV infection, and 68 patients (45.94%) had stage 3 HIV infection at the time of switch.

It was discovered that in the study group, abnormal ALT and AST values were present in 35 cases (23.64). Regarding the metabolic profile, 49 cases (33.10%) had abnormal triglyceride levels, which affected both genders almost equally (18.24% men and 14.86% women). Cholesterol blood levels were elevated in 54 cases at baseline (Table 2). 

### 3.2. Antiretroviral Regimens

**Figure 1:**
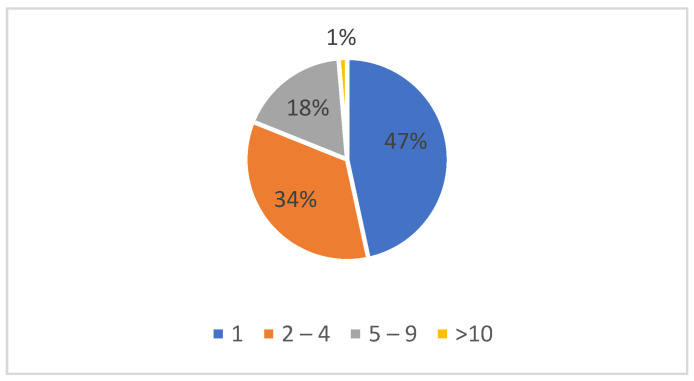
Number of antiretroviral regimens before the present switch.

Some of the study group cases had multiple antiretroviral treatment drug associations (regimens) before the present switch. Almost half of the patients were at their first ARV treatment change—69; 51 cases had between 2 and 4 regimens, 26 cases had between 5 and 9 regimens, and 2 cases had more than 9 antiretroviral therapy regimens (Figure 1). 

ART switch was made according to the Liverpool drug–drug interaction checker, to avoid drug–drug interactions if the patient was taking other co-medication.

Before the ART switch, the patients had a cardiological (106 cases, 72%), neurological (82 cases, 55.33%), internal medicine (51 cases, 34.66%), psychiatric (15 cases, 10.13%), and gastroenterological (20 cases, 13.33%) check-up.

### 3.3. Switching Antiretroviral Therapy Criteria

**Figure 2:**
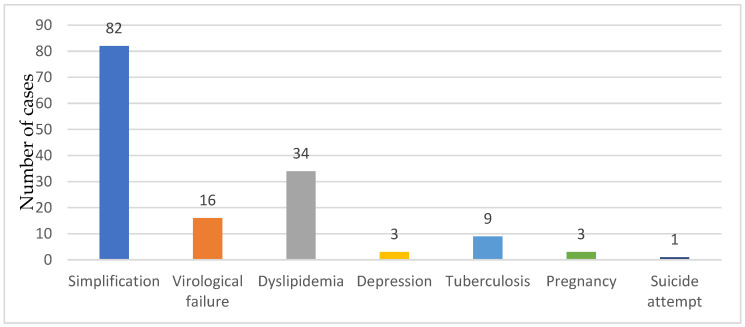
The reasons for ART switch.

The main reason for the ART switch was the simplification of the ART regimen (82 cases, 55.40%), viro-immunological failure (16 cases, 10.66%), side effects correlated to the ART regimen, dyslipidemia (34 cases 22.97%), depression (3 cases, 2.02%), suicide attempt (1 case, 0.67%), pregnancy (3 cases 2.02%) and tuberculosis (9 cases, 6.08%) (Figure 2).

ART before the switch was represented by protease inhibitors (PI) that accounted for 84 cases (56.75%) of the ART switch, followed by non-nucleoside reverse transcriptase inhibitor (NNRTI) regimens (37 cases, 25%), integrase inhibitor (INSTI) regimens using more than one pill (13 cases, 8.78%), and other regimens (14 cases, 9.45%) (Table 3). 

**Figure 3:**
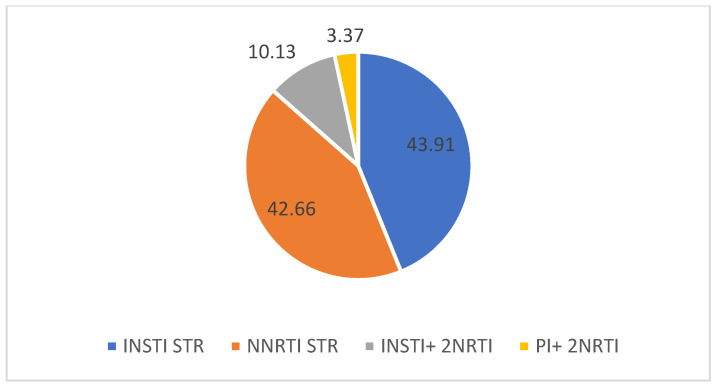
ARV regimens selected after the therapy switch (%).

Following the therapy switch, integrase inhibitor-based ART single-tablet regimens accounted for 65 cases, at 43.91% (from which Bictegravir/Emtricitabine/tenofovir Alafenamide accounted for 38 cases, 25.67%, and Dolutegravir/ Lamivudine 27 cases, 18.24%), with non-nucleoside reverse transcriptase inhibitor single-tablet regimens coming in second, in 63 cases (42.66%), followed by other regimens (integrase inhibitors + 2 NRTI MTR- 15 cases, 10.13%, Protease inhibitors+ 2 NRTI MTR, 5 cases, 3.37%) (Figure 3).

**Figure 4:**
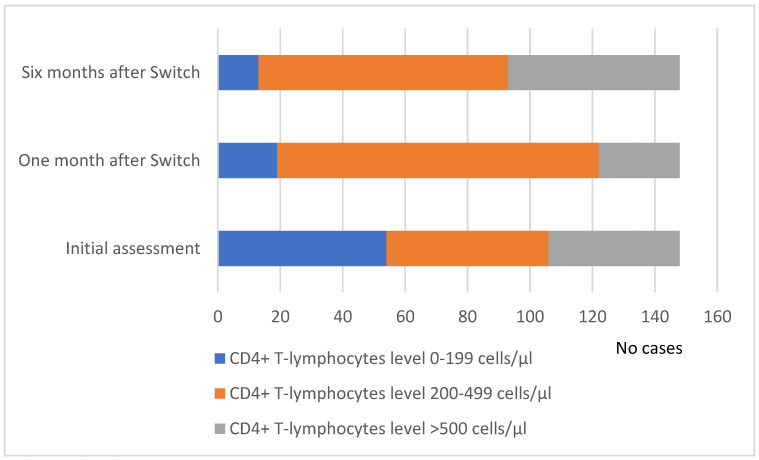
Evolution of the CD4+ T cell number distribution at baseline and one and six months after the ART switch.

The patients were evaluated one month after the ART switch, and the viro-immunological condition showed a significant decrease in HIV viremia, with an increased level of CD4+ T cells. As a result, 103 patients (69.59%) had a value between 200 and 499 cells/μL, 26 patients (17.50%) had a value exceeding 500 cells/μL, and 19 patients (12.83%) had a CD4 value below 200 cells/μL. The majority of patients, regardless of gender, exhibited a CD4+ T-lymphocyte count of 200–499 cells/μL or above (Figure 4).

**Figure 5:**
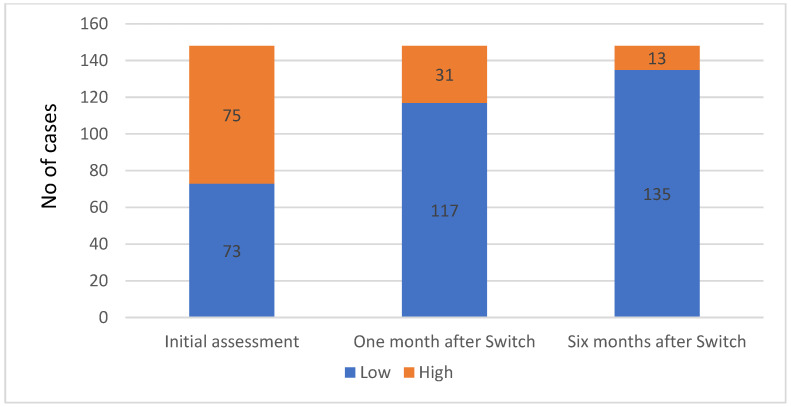
HIV viral load status distribution at baseline and one and six months after the ART switch.

The HIV viral load decreased after starting antiretroviral therapy, with viral suppression happening in 55.26% of cases (21 cases). The patients who had undetectable viremia during the first evaluation were either transferred from another regional HIV/AIDS center or had received their diagnosis overseas and had begun antiretroviral therapy (ART) at the time of their initial evaluation at our center (Figure 5).

At the six-month evaluation, abnormal ALT and AST values were present in 13 cases (8.78%). Regarding the metabolic profile, 27 cases had abnormal triglyceride levels, which affected both genders almost equally (10.13% men and 8.10% women). Additionally, elevated blood cholesterol levels were found in 36 cases, 24.32%) (Table 2). 

## 4. Discussion

Antiretroviral therapy has revolutionized the management of HIV infection, significantly improving the quality of life and life expectancy of individuals living with this chronic disease. 

ART switch with an individualized therapy offers a promising approach to optimizing HIV treatment outcomes. By focusing on viral suppression and immune reconstitution, addressing treatment challenges, and exploring novel ARV agents, we aimed to observe the evolution of the study group after the ART switch so that we can improve the overall health and well-being of individuals living with HIV.

In our cohort, the ART switch occurred more frequently in men, in younger patients, and in the 30–39-year-old group. Similar findings were revealed by a team of authors analyzing the antiretroviral therapy switch rate and switching pattern for people living with HIV from a national database in Japan [3]. Simplification was shown, as switching to a single daily tablet regimen is probably better received by this demographic category, with an active, busy lifestyle.

The CD4 T lymphocytes and HIV viremia load one month after the switch were favorable, obtaining viral suppression in some cases that had detectable viremia before the switch. Furthermore, the adherence and compliance of the patients to the ART were observed because the new switch regimens were mostly single-tablet regimens.

However, challenges such as drug resistance, treatment toxicity, and long-term tolerability remain, necessitating the exploration of innovative treatment strategies. Among these approaches, ART switch—an intentional change from one ARV regimen to another—has emerged as a promising experimental therapy in the field of HIV management [16,17].

The primary goal of ART switch is to optimize viral suppression, enhance immune reconstitution, and minimize treatment-associated complications. Previous studies [18,19] found that ART switch strategies have the potential to significantly reduce the viral load in HIV patients, leading to improved clinical outcomes. This is particularly crucial in cases of virological failure on current regimens, where a switch to alternative ARVs can offer a fresh chance at viral suppression. We agree with this statement. We observed that patients had a significantly improved virological status after the ART switch.

In addition to virological benefits, ART switch has been associated with notable improvements in immunological parameters, including increases in CD4 cell counts following the ART switch, indicating enhanced immune reconstitution. This immune recovery is crucial for bolstering the body’s defenses against opportunistic infections and disease progression in HIV patients [20,21]. At the one- and six-month evaluation after the ART switch, many of our patients showed significant increases in their CD4 cell counts.

Despite the potential advantages of ART switch, challenges in treatment adherence often arise. Several studies highlighted the importance of adherence support programs, as many patients reported difficulties in maintaining consistent medication routines post-switch. Adherence remains a cornerstone of successful HIV treatment, emphasizing the need for comprehensive patient education and support services [22,23]. We observed that in our patients, pill burden was the major factor correlated with adherence. 

Comparative effectiveness studies have delved into the various ART switch strategies used to identify optimal approaches. Lodi et al. conducted a network meta-analysis comparing the efficacy of different switch regimens, providing valuable insights for clinicians. This research highlighted the importance of tailoring switch strategies to individual patient needs and treatment histories [24]. It is important to ponder the benefits and risks of ART switch. We need more studies regarding the outcome of ART switch.

While ART switch holds promise as an experimental therapy, the consideration of safety profiles is paramount. A study [25] that investigated the safety outcomes of different ART switch regimens shed light on their potential risks, such as cardiovascular events and metabolic disturbances. Clinicians must weigh the benefits against the potential adverse effects when making switch decisions. 

Special attention must be given to women of childbearing age, and in this situation, the guidelines offer the right recommendation [26,27]. Dyslipidemia is associated with increased cardiovascular disease and cognitive decline [28,29]. Other studies have shown that PI-based therapy has deleterious effects on lipid metabolism [30,31]. We also observed that protease inhibitors increase metabolic disorders such as dyslipidemia, this being one of the reasons for ART switch. At the six-month evaluation after the switch, patients showed decreased values of triglycerides and cholesterol, and also increased hepatic enzyme levels.

The change in antiretroviral therapy among HIV-infected adults from the Regional HIV/AIDS Center in NE Romania during the COVID-19 pandemic was carried out with the careful supervision of both the clinical-biological and viro-immunological evaluation parameters, and in the context of psychological counseling. 

In our country, as a single-tablet regimen, the most affordable is Doravirine/lamivudine/tenofovir disopoxil fumarate, with Bictegravir/emtricitabine/tenofovir alafenamide at the opposite pole. The ART regimens are being supported by the national program currently in Romania, making it easier to access the new ART co-formulated single-tablet regimens.

This study has several limitations regarding the limited area that contains the study group, the northeastern part of Romania. It would be more significant if we could include the other regions of our country. Difficulties related to the quantification of the patient’s adherence to treatment could interfere with the interpretation of the reasons that led to the ART switch.

ART switch, as an individualized therapy in people living with HIV, offers a multifaceted approach to treatment optimization. Through optimizing viral suppression, enhancing immune reconstitution, and addressing treatment challenges, ART switch has the potential to improve the long-term outcomes and quality of life for individuals living with HIV. However, it requires careful patient monitoring, adherence support, and the consideration of safety profiles to ensure its effectiveness. In the future, we aim to include the other regions of our country to better understand the impact of ART switches on the lives of people living with HIV. It is crucial to continue routinely monitoring the virologic efficacy and safety parameters to look for any new adverse events connected to ART modification and drug–drug interactions. Many of the newer medicines tested for ART switches allow patients to stay on these regimens for many years without having to switch again because of their decreased pill burden, frequency of administration, and similar if not improved virologic efficacy, safety, and tolerability.

## 5. Conclusions

In our study group, the ART switch occurred more frequently in young male adults, the most common reason being treatment simplification involving novel ARV agents in order to improve the overall health and well-being of individuals living with HIV. Most responded well to the change in the treatment, with improved CD4 counts, decreased viral loads and a better lipid and hepatic enzyme profile. 

ART switch, as an individualized therapy, offers a promising approach to optimizing HIV treatment outcomes. The integration of personalized treatment approaches, safety considerations, patient education, and support programs will be crucial in maximizing the benefits of ART switch therapies.

## Figures and Tables

**Table 1:** Distribution of the study group by age.

	**With Switch**		**Without Switch**		***p***
**Age (Years)**	**n (148)**	**%**	**n (1562)**	**%**	
20–29	20	13.5	260	16.6	*p* < 0.00001
30–39	85	57.4	473	30.3	
40–49	19	12.8	387	24.8	
50–59	17	11.5	344	22.0	
Over 60	7	4.8	98	6.3	

**Table 2:** Distribution of cases according to sex, metabolic disorder, and liver enzyme levels.

Laboratory Marker	Value	Before Switch		After Switch		
		N (148)	%	N (148)	%	*p*
ALT	normal	113	76.4	135	91.2	0.0005
	elevated	35	23.6	13	8.8	
AST	normal	113	76.4	135	91.2	0.0005
	elevated	35	23.6	13	8.8	
GGT	normal	117	79.1	131	88.5	0.028
	elevated	31	20.9	17	11.5	
Total cholesterol	normal	94	63.5	112	75.7	0.023
	elevated	54	36.5	36	24.3	
HDL-col	normal	101	68.24	112	75.67	0.15
	elevated	47	31.75	36	24.32	
LDL-col	normal	99	66.89	112	75.67	0.095
	elevated	49	33.10	36	24.32	
Triglycerides	normal	97	65.5	121	81.75	0.001
	elevated	51	34.5	27	18.24	

**Table 3:** HIV/AIDS cases by ART regimen before the present switch.

ART Regimen	N (148)	%
Protease inhibitors + 2 NRTI (more than one pill)	84	56.7
Non-nucleoside reverse transcriptase inhibitor + 2 NRTI (more than one pill)	37	25
Integrase inhibitors + 2 NRTI (more than one pill)	13	8.7
Other	14	9.45

## Data Availability

All data generated or analyzed during this study are included in this published article.
